# Efficient taxa identification using a pangenome index

**DOI:** 10.1101/gr.277642.123

**Published:** 2023-07

**Authors:** Omar Ahmed, Massimiliano Rossi, Christina Boucher, Ben Langmead

**Affiliations:** 1 Department of Computer Science, Johns Hopkins University, Baltimore, Maryland 21218, USA;; 2 Department of Computer and Information Science and Engineering, Herbert Wertheim College of Engineering, University of Florida, Gainesville, Florida 32611, USA

## Abstract

Tools that classify sequencing reads against a database of reference sequences require efficient index data-structures. The *r*-index is a compressed full-text index that answers substring presence/absence, count, and locate queries in space proportional to the amount of distinct sequence in the database: O(r) space, where *r* is the number of Burrows–Wheeler runs. To date, the *r*-index has lacked the ability to quickly classify matches according to which reference sequences (or sequence groupings, i.e., taxa) a match overlaps. We present new algorithms and methods for solving this problem. Specifically, given a collection D of *d* documents, $D=\{T_{1},T_{2},\ldots ,T_{d}\}$ over an alphabet of size σ, we extend the *r*-index with O(rd) additional words to support document listing queries for a pattern S[1..m] that occurs in ndoc documents in D in $O(m\log\;\log_{w}(\sigma +n/\;r)+ndoc)$ time and O(rd) space, where *w* is the machine word size. Applied in a bacterial mock community experiment, our method is up to three times faster than a comparable method that uses the standard *r*-index locate queries. We show that our method classifies both simulated and real nanopore reads at the strain level with higher accuracy compared with other approaches. Finally, we present strategies for compacting this structure in applications in which read lengths or match lengths can be bounded.

Metagenomic read (Wood et al. 2019) classification allows researchers to study organisms present in an environmental sample. Tools like Kraken 2 (Wood et al. 2019) and Centrifuge (Kim et al. 2016) accomplish this using an index of the reference sequences. Kraken 2 (Wood et al. 2019) builds a compact hash table that maps minimizer sequences onto the taxonomic lowest-common ancestor of the genomes in which it occurs. Centrifuge (Kim et al. 2016) uses an FM-index (Ferragina and Manzini 2000) to find substring matches that are combined to make classification decisions. But as databases of reference sequences continue to grow, these tools encounter difficulties with scaling and accuracy. Nasko et al. (2018) showed that the specificity of *k*-mer-based approaches like Kraken 2 can suffer as the reference database (i.e., RefSeq) grows, because the addition of new sequences causes more *k*-mers (or minimizers) to co-occur in distant parts of the taxonomy. The FM-index at the core of Centrifuge does not naturally scale to pangenomes; rather, it requires an initial work-intensive step that compresses the genomes in a way that elides some of the underlying genetic variation.

The *r*-index (Gagie et al. 2020) is a successor to the FM-index that indexes repetitive texts using O(r) space, where *r* is the number of runs in the text's Burrows–Wheeler transform (BWT). Because *r* grows only with the amount of *distinct* sequence in the collection, the *r*-index scales naturally to large pangenomes and reference databases like the ones used for taxonomic classification. Because it is a full-text index, the *r*-index can find matches of any length, unconstrained by a particular choice of *k*-mer length.

Although the *r*-index has already been applied to pangenomic pattern matching (Kuhnle et al. 2020; Rossi et al. 2022) and binary classification (Ahmed et al. 2021), it has so far lacked the ability to solve multiclass classification problems in an accurate and efficient manner. A straightforward approach would be to use a standard backward search in the *r*-index and then use locate queries to locate the offsets in the concatenated text in which the pattern occurs. These offsets can then be cross-referenced with another structure to determine which documents they occur in. This requires an amount of work proportional to the number of occurrences *occ*, which is expensive, particularly for repetitive matches against a pangenome.

We hypothesized that extending the *r*-index to multiclass classification could be accomplished by augmenting it with efficient facilities for *document listing*, namely, the ability to report all the reference sequences (documents) in which a particular pattern occurs. A document, which we will sometimes call a “class,” could consist of a single genome or a collection of related genomes.

An early study by Muthukrishnan (2002) described a specialized index for document listing consisting of a generalized suffix tree and a document array. It provided O(m+ndoc) queries, where *m* is the length of the pattern, and *ndoc* is the number of distinct documents in which it occurs. But this came at the cost of O(nlogn) bits of space, where *n* is the total length of the texts, which is impractical for large pangenome databases. Sadakane (2007) improved on this by introducing a new succinct document array representation and building on succinct representations of suffix trees and arrays. He showed how to reduce the index size to |CSA|+O(n) bits, where |*CSA*| is the size of the compressed suffix array using statistical compression with an increased time complexity of O(m+ndoc⋅logn), a high cost for repetitive text collections (Cobas and Navarro 2019). Later efforts further reduced the required space using grammar compression (Cobas and Navarro 2019) and relative Lempel–Ziv compression (Puglisi and Zhukova 2021).

We present a new method that solves the document listing problem in $O(m\log\;\log_{w}\sigma +ndoc)$ time and O(rd) space using the *r*-index. Importantly, we also show how to use the prefix-free parsing process to build the profile simultaneously with the BWT. This document array structure (an example is shown in Table 1) can be sampled and stored at the run boundaries of the BWT, yielding a space complexity of O(rd). At query time, after performing a backward search for a pattern, we can report the document listing by simply examining the current document array profile (exemplified in Table 2), which is an array of *d* integers, as opposed to performing a query for each occurrence of a pattern. We also discuss practical optimizations that can be used to reduce the space usage of this data-structure even further in the context of metagenomic read classification. In our evaluations, we compare the query time and index size for our approach to an alternative that uses the standard *r*-index locate query to report document listings. Furthermore, we attempt to classify different strains of *Escherichia coli* and *Salmonella enterica* using our document array profiles in comparison to using SPUMONI 2's sampled document array (Ahmed et al. 2023). Finally, we believe that our theoretical guarantees will prove useful for the community by allowing read classification to be compared in a grounded manner that complements practical evaluation.

## Results

We performed all the experiments on an Intel Xeon gold 6248R 32-core processor running at 3.00 GHz with 1.59 TB of RAM with 64-bit Linux. Time was measured using the std::chrono::system_clock from the C++ standard library. Our source and experimental codes can be found at GitHub (see Software availability). The *r*-index code used in our experiments can be found at GitHub (https://github.com/maxrossi91/r-index).

### Comparing the query time and index size

To assess the speed of document listing, we compared the query time for the document array profiles to the query time for locate queries using the *r*-index. We attempted to compare our solution to the method of Cobas and Navarro (2019); however, we ran into various run-time errors when using it as described, so we were not able to include it in the results.

We built a series of indexes over genomes from different collections of bacterial species, described in Table 3. We simulated nanopore sequencing reads using PBSIM2 (Ono et al. 2021) at 95% read accuracy. We then used MONI (Rossi et al. 2022) to query each read against the pangenome index, extracting a total of 1 million maximal exact matches (MEMs) for each class.

**Figure 1.**
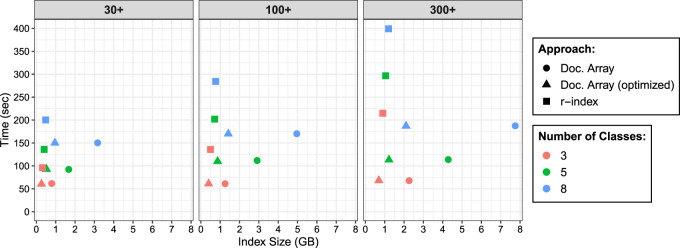
Query time and index size when performing document listing queries using the document array profiles and the *r*-index. We varied the size of the database increasing from 30 bacterial genomes to 300 bacterial genomes. For each species/class, we would simulate nanopore reads at 95% accuracy and extract 1 million maximal-exact matches (MEMs) to query the data-structures. Therefore, for the three-class, five-class, and eight-class indexes, we queried them with 3 million, 5 million, and 8 million MEMs, respectively. This explains why the query time would increase for the indexes containing more classes.

We tested two variants of the document array profile data-structure. The first (labeled “Doc. Array” in Fig. 1) uses the standard document array profile, in which the width of each profile entry requires $\left\lceil \log_{2}(|S|)\right\rceil$ bits. The second (labeled “Doc. Array (optimized)”) instead stores truncated lcp values, so that lcps greater than 255 are stored as 255, so that only $\lceil \log_{2}(255)\rceil =8$ bits are required per entry. This optimization is appropriate in real-world situations in which either the reads are known to be short (e.g., Illumina sequencing reads) or we would otherwise expect MEMs longer than 255 to be rare.

We observed that the query time using document array profiles was faster than the *r*-index locate query. For the three-class database, the document array profiles ranged from 1.6–3.2 times faster. As more genomes were added to the database, the query time for the three-class *r*-index increased by 2.2-fold (214.87 sec vs. 96.2 sec), whereas query time for the document array profile was essentially constant (67.8 sec vs. 61.7 sec). This shows a key advantage of our document listing; unlike when using the *r*-index locate queries, our query time is independent of the number of pattern occurrences.

We noted that the size of the *r*-index stayed relatively constant as the number of classes increased. However, for the document array profile (both standard and optimized), the index size grew with the number of classes, consistent with its O(rd) space complexity. As an example, in the “30+” genome database, focusing on the standard document array, the eight-class document array was 2.32 times larger than the five-class document array. Because *d* increased by 1.67 times and *r* increased by 1.43 times (79,722,710 vs. 55,559,459), we therefore would expect to see an index increase of about 2.39 times (1.67 × 1.43), which is close to what we see in practice (2.32).

We also observed for the three-class, “30+” genome database, the optimized document array was smaller than the *r*-index. The *r*-index stores a run-length encoded BWT (RLEBWT) along with the suffix array sampled at run boundaries in the BWT where each sample is stored in 5 bytes. The optimized document array also stores a RLEBWT; however instead of the suffix array, it replaces it with the document array profiles. Because it is a three-class database, each profile sampled at the run boundaries will only consist of 3 bytes, which explains why, overall, the optimized document array is smaller than the r-index for those conditions.

Additionally, as expected, the optimized document array profile was smaller than the standard profile; for the 300-genome database, it was 3.3 times smaller. We suggest further optimizations to reduce the document array profile size in the Discussion below.

### Species and strain-level classification

We hypothesized that the document array profiles could particularly improve read classification accuracy in difficult scenarios in which it is important to be able to list all documents for each MEM. We compared the performance of the document array profile to another tool and structure designed for read classification: SPUMONI 2's (v2.0.0) (Ahmed et al. 2023) sampled document array. SPUMONI 2's sampled document array is quite simple; for each BWT run boundary, it simply converts the suffix array position to the document number in which that position occurs. Using these document labels, it is capable of reporting one document in which a particular exact match occurs. This is sufficient in situations in which reads contain many distinct matches (e.g., MEMs), so that document information can be pooled across the various matches to come to an overall conclusion. But in situations in which the documents are very similar to each other or in which reads are short or have a high error rate, we expect the full document array profile to impart higher accuracy.

**Figure 2.**
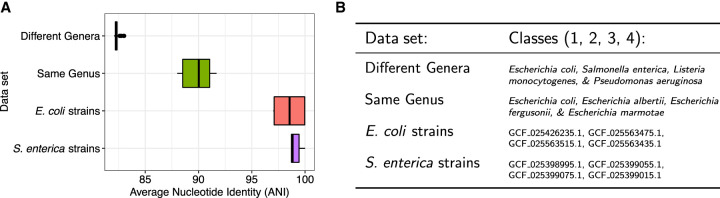
Summary of reference data sets of increasing difficulty for read classification. (*A*) Sequence homology, measured as average nucleotide identity (ANI) for all across-class pairs of sequences. ANI was estimated with fastANI (Jain et al. 2018). (*B*) List of the specific species and strains used for classes 1, 2, 3, and 4 for each of the four data sets. In the case of “different genera” and “same genus,” we used 10 genomes per class. In the case of “*E. coli* strains” and “*S. enterica* strains,” we used a single genome for each strain.

We tested the two structures on increasingly difficult data sets, with each data set consisting of reference genomes from four distinct classes (Fig. 2). We used PBSIM2 (Ono et al. 2021) to simulate 50,000 nanopore reads from each class at 95% accuracy and then classified the reads using both document array approaches. Specifically, we identified all MEMs between the reads and the pangenome index, filtering to just MEMs of length 15 or longer. We then used the different document structures to obtain matching documents for each MEM: In the case of SPUMONI 2, we retrieved one document per MEM; in the case of our document array profile, we retrieved all documents where the MEM occurred. We then weighted the documents according to the length of the MEM and assigned each read to a document according to which received the largest total weight across all reported MEM/document combinations.

**Figure 3.**
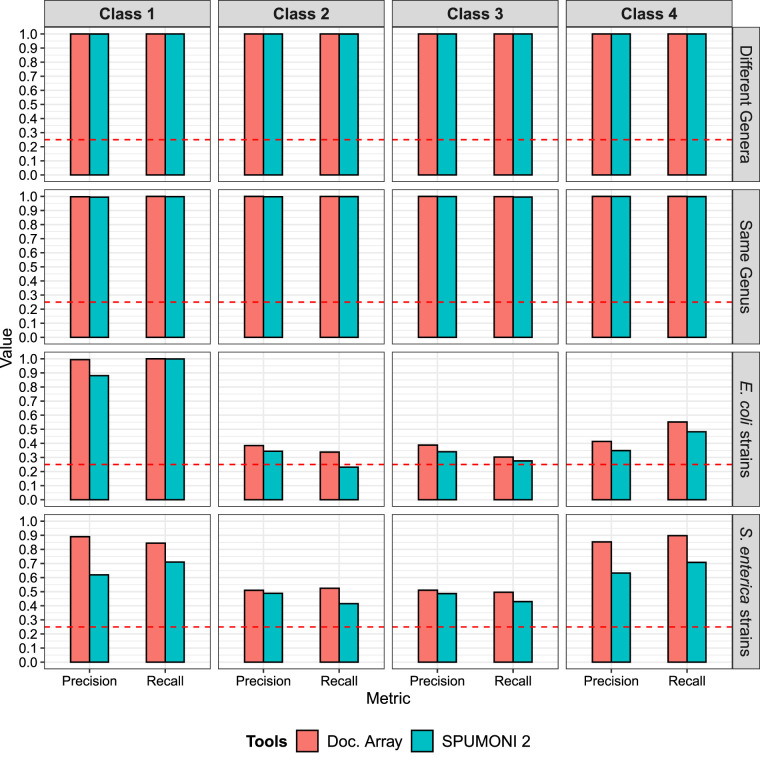
Classification results using the document array profiles and SPUMONI 2's (Ahmed et al. 2023) sampled document array across four different data sets each with the four classes described in Figure 2.

We observed that when the data set consisted of classes with low between-class sequence similarity (“different genera” and “same genus”), both methods performed well, with low classification errors (Fig. 3). However, for data sets with high sequence similarity (>97.5% ANI), such as the “*E. coli* strains” and “*S. enterica* strains,” we see that the full document array profile provided greater classification accuracy compared with SPUMONI 2's one-document-per-match approach.

### Classification using real nanopore mock community reads

We extended our analysis to real sequencing reads. We used nanopore reads from the UNCALLED (Kovaka et al. 2021) paper, which performed Oxford Nanopore sequencing of a Zymo mock community consisting of eight species (*Staphylococcus aureus*, *S. enterica*, *E. coli*, *Pseudomonas aeruginosa*, *Listeria monocytogenes*, *Enterococcus faecalis*, *Bacillus subtilis*, and *Saccharomyces cerevisiae*). We extracted a set of 582,042 reads from the data set that uniquely mapped to one of the seven bacterial species using minimap2 (Li 2018). We shortened each read to 2000 bp.

**Figure 4.**
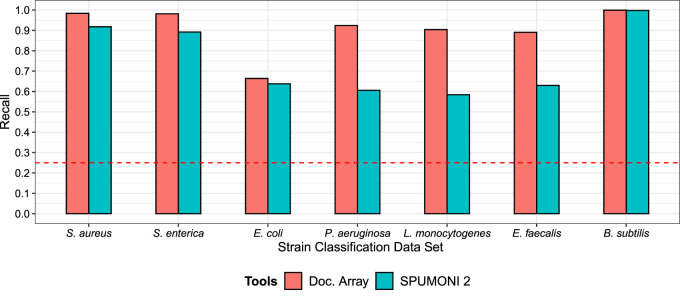
Comparing the document array profiles and SPUMONI 2's (Ahmed et al. 2023) sampled document array on seven different strain-level classification tasks using real nanopore reads from the UNCALLED (Kovaka et al. 2021) project.

For each bacterial species, we constructed a database comprising of four strains from that species, one of which was chosen to be the actual strain used for the Zymo mock community. The other three strains were obtained from RefSeq. We then compared the strain-level classification accuracy of the two document array structures using the same MEM-weighted approach as was used in the previous experiment. As in the previous experiment, we observed that the document array profile enabled more accurate strain-level read classification (Fig. 4). This was true for reads derived from all seven of the bacterial species (though both approaches had near-perfect recall for *B. subtilis* reads).

## Discussion

We described a new data-structure called the document array profile, along with an efficient algorithm for building the structure simultaneously with a pangenome *r*-index. This structure enables tools to find exact matches with respect to a full-text pangenome index while simultaneously learning which reference sequences the matches belong to. This opens the door to new applications of pangenome indexes, including in metagenomics read classification.

The structure requires O(rd) space and can compute a full document listing for a match in $O(m\log\;\log_{w}\sigma +ndoc)$ time. We showed that, as the pangenome database grows in size, the document array profile's speed advantage grows relative to the standard *r*-index and its locate queries. Further, we showed that the structure's ability to list all documents associated with a match enables greater accuracy compared with an existing alternative that considers only one document per match.

The main weakness of the document array profile is the fact that its space usage grows linearly with the number of documents *d*. This makes it difficult for it to be used in scenarios with a large number of documents (classes), which is the case in taxonomic read classification, in which there are thousands of species. However, this data-structure can be optimized even further to reduce its space usage with domain-specific knowledge. For example, in sequencing read classification, an exact match shorter than 15 bases might be too nonspecific to be helpful for classification. In that case, each element of the document array profile could be made “sparse,” consisting only of values greater than 14.

An additional optimization would be to adopt a “top *k*” strategy. That is, rather than store lcp values to all possible documents, we can restrict the structure to store only the lcp values to the *k* documents having the greatest lcp at the run boundary. This allows us to bound the size of the structure while retaining the strongest match-to-document associations.

Recently, Cobas et al. (2020) designed solutions to the *document listing with frequencies* problem using the *r*-index as the text index. This problem is a more difficult task because it requires reporting not only the document listing but also the frequency of the pattern in each document. The frequency information could add valuable data for taxonomic classification because it gives an indication if a pattern is “common” within a document or if it is rather rare. Future work on the document array profiles will consist of exploring the possibility integrating elements of solution (Cobas et al. 2020) to allow the document array profiles to report frequencies along with the document listing.

## Methods

### Preliminaries

A string *S*[1..*n*] of length |*S*| = *n* is the concatenation of characters *S*[1] · · · *S*[*n*] drawn from an alphabet Σ of size σ. We denote by ε the *empty* string that is the only string of length 0. We assume *S* is terminated by a special symbol $∉Σ lexicographically smaller than all symbols in Σ. Given two integers 1 ≤ *i*, *j* ≤ *n*, we denote with *S*[*i*..*j*] = *S*[*i*] · · · *S*[*j*] the *substring* of *S* spanning positions *i* through *j* if *i* ≤ *j*, and S[i..j]=ε otherwise. Given two integers 1 ≤ *i*, *j* ≤ *n*, we refer to *S*[*i*..*n*] as the *i*th *suffix* of *S* and to *S*[1..*j*] as the *j*th *prefix* of *S*. Given two strings *S*[1..*n*] and *T*[1..*m*], we denote with lcp(*S*, *T*) the length of the *longest common prefix* of *S* and *T*.

### Suffix array, inverse suffix array, and longest common prefix array

Given a string *S*, the *suffix array* (Manber and Myers 1993) SA_*S*_[1..*n*] is the permutation of {1, …, *n*} that lexicographically sorts the suffixes of *S*. The *inverse suffix array* ISA_*S*_[1..*n*] is the inverse permutation of SA_*S*_[1..*n*]; that is, for all *i* = 1, …, *n*, SA_*S*_[ISA_*S*_[*i*]] = *i*. The *longest common prefix* array LCP_*S*_[1..*n*] stores the length of the longest common prefix between lexicographically consecutive suffixes of *S*; formally, LCP[1] = 0, and for all *i* = 2, …, *n*, LCP[*i*] = lcp(*S*[SA_*S*_[*i* − 1]..*n*], *S*[SA_*S*_[*i*]..*n*]).

### Burrows–Wheeler transform

Given a string *S*, the *Burrows–Wheeler transform* (Burrows and Wheeler 1994) BWT_*S*_[1..*n*] is the reversible permutation of *S* defined as the last column of the matrix of the lexicographically sorted rotations of *S*. When *S* is terminated by $, we can define for all *i* = 1, …, *n*, BWT_*S*_[*i*] = *S*[SA_*S*_[*i*] − 1], where *S*[0] = *S*[*n*]. The LF*-mapping* is the permutation of {1, …, *n*} that maps every character in the BWT_*S*_ to its predecessor in text order; formally, $\mathrm{LF}[i]=\mathrm{IS}A_{S}[(SA_{S}[i]-2\;\mathrm{mod}\;n)+1]$. We define *r* as the number of maximal equal-letter runs of BWT_*S*_. When clear from the context, we refer to SA_*S*_, ISA_*S*_, LCP_*S*_, and BWT_*S*_ as SA, ISA, LCP, and BWT, respectively.

### *r*-Index

The *r*-index (Gagie et al. 2020) is a text index that stores the run-length encoded BWT and the SA entries sampled at run boundaries. Given a text *S*[1..*n*] and a pattern *P*[1..*m*], the *r*-index allows you to find all occurrences of *P* in *S* in $O(m\log\;\log_{w}(\sigma +n/\;r)+occs\log\;\log_{w}(n/\;r))$ and O(r) words of space, where *occs* is the number of occurrences of *P* in *S*. This result was later improved to $O(m\log\;\log_{w}(\sigma )+occs)$ (Nishimoto et al. 2022).

### Document array

We denote with $D=\{T_{1},\ldots ,T_{d}\}$ the *collection* of documents (strings)*T*_1_, …, *T*_*d*_, and we denote with $T[1..n]=T_{1}\cdots T_{d}$ the concatenation of the documents. The *document array* (Muthukrishnan 2002) DA[1..*n*] stores for each position *i* the document index of $T[SA_{T}[i]..n]$. An important problem in document retrieval is the *document listing* problem.

Problem 1. *Given a collection* $D=\{T_{1},\ldots ,T_{d}\}$ *and a pattern P, return the set of documents* L⊆D *where P occurs*.

### Supporting document listing on the *r*-index

Given the text T that is the concatenation of the documents of D such that T has length *n*, let BWT be the BWT of T that has *r* equal-letter runs.

Definition 1. *For all positions* 1 ≤ *i* ≤ *n in the* BWT *of* T, *we define the* profile of the document array *as the array P*_DA_[*i*][1..*d*] *that stores for each position j* = 1, …, *d the length of the longest common prefix between* T[SA[i]..n] *and all suffixes of document T*_*j*_*. Formally,*

$$P_{\mathrm{DA}}[i][j]=\max\{\mathrm{lcp}(T[\mathrm{SA}[i]..n],T[\mathrm{SA}[k]..n])|1\leq k\leq n\;\mathrm{and}\;\mathrm{DA}[k]=j\}.$$

Lemma 1. Given the BWT *of* T *and the profile of the document array P*_DA_*, for all pairs* (*i*, ℓ) *of positions and lengths corresponding to a substring* S=T[SA[i]..SA[i]+ℓ−1]*, we can find the list of ndoc documents where S occurs in* T *in* O(d) *time*.

Proof. By definition of *P*_DA_[*i*], we have that *S* occurs in document *T*_*j*_ if and only if ℓ ≤ *P*_DA_[*i*][*j*]. Hence, we can scan the profile of the document array in position *i*. For all documents *j* = 1, …, *d*, we check if the length of the substring is less than or equal to the value stored in the profile for the *j*th document. This requires one comparison per document, or O(d) time.

Example 1. *In the example in* Table 1*, if we look at P*_*DA*_[4] = [1, 2, 4] *corresponding to the suffix* AAC#*, we have that (1) for the pair* (21, 1) *the substring S* = *A occurs in documents* 1*,* 2*, and* 3*, because all values of P*_*DA*_[4] *are not smaller than* 1*;* (*2) for the pair* (21, 2) *the substring S* = *AA occurs in documents* 2*, and* 3 *because* 2 > *P*_*DA*_[4][1]*; (3) for the pair* (21, 3) *the substring S* = *AAC occurs only in document* 3 *because* 3 *is greater than both P*_*DA*_[4][1] *and P*_*DA*_[4][2].

If we store each entry of the *profile of the document array P*_DA_[*i*] as a list of sorted pairs (*P*_DA_[*i*][*j*], *j*), the query time can be reduced to O(ndoc) by simply scanning the list of pairs from the document with the largest profile value to the first document that has a profile value smaller than ℓ.

**Table 1.**
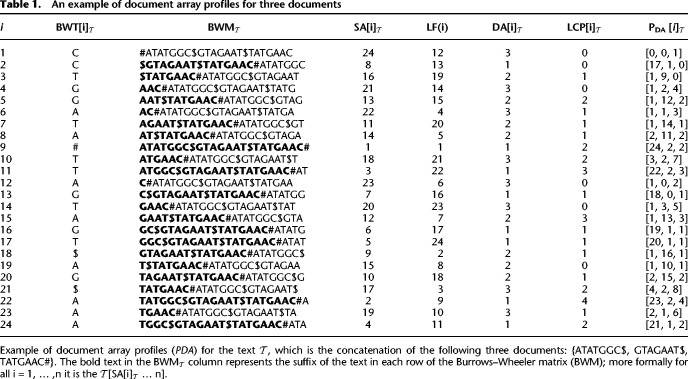
An example of document array profiles for three documents

**Table 2.**
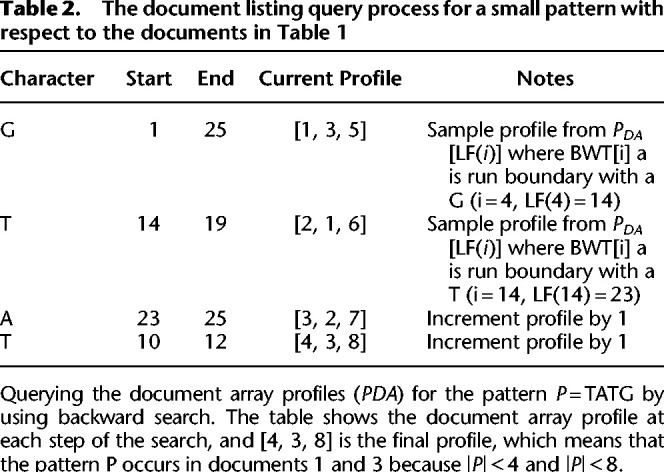
The document listing query process for a small pattern with respect to the documents in Table 1

**Table 3.**
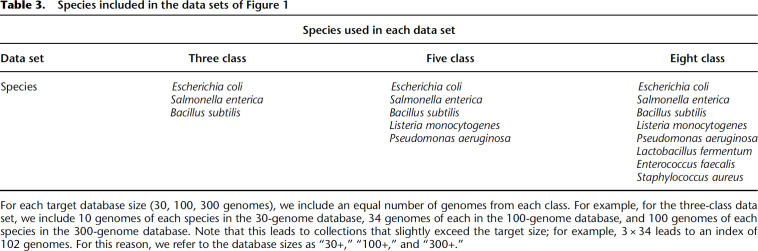
Species included in the data sets of Figure 1

### Sampling the profile of the document array

Storing the entire profile of the document array requires O(nd) words of space, which will be excessive for pangenomes. We seek to compress the profile of the document array by sampling it similarly to how *r*-index samples the suffix array.

Let BWT[*s*..*e*] be a maximal equal-letter run of the BWT of T. We store in position *s* and *e* the entries of the profile of the document array in positions LF(*s*) and LF(*e*), respectively. Applying the same reasoning as the toehold lemma (Policriti and Prezza 2016), we can show that this is enough to recover the document listing for a query pattern *S*.

The first property of the profile of the document array that we show is an upper bound on the values of the profile, when performing an LF step.

Lemma 2. *For all positions* 1 ≤ *i* ≤ *n in the* BWT *of* T *such that* DA[*i*] = DA[LF(*i*)]*, for all j* = 1, …, *d*, *it holds that P*_DA_[LF(*i*)][*j*] ≤ *P*_DA_[*i*][*j*] + 1.

Proof. From the definition of *P*_DA_[*i*][*j*], there exists a position 1 ≤ *k* ≤ *n* such that

$$\begin{array}{rl} \mathrm{lcp}(T[\mathrm{SA}[i]..n],T[\mathrm{SA}[k]..n]) & \geq \max\{\mathrm{lcp}(T[\mathrm{SA}[i]..n],T[\mathrm{SA}[k^{\prime }]..n])|\\ 1 & \leq k^{\prime }\leq n\;\mathrm{and}\;\mathrm{DA}[k^{\prime }]=j\}. \end{array}$$

Hence, if we consider the character preceding SA[*i*], that is, BWT[*i*], then by maximality of *k*, we have that

$$\begin{array}{rl} & \max\{\mathrm{lcp}(T[\mathrm{SA}[i]-1..n],T[\mathrm{SA}[k^{\prime }]..n])|1\leq k^{\prime }\leq n\;\mathrm{and}\;\;\mathrm{DA}[k^{\prime }]=j\}\\ & \;\leq \mathrm{lcp}(T[\mathrm{SA}[i]..n],T[\mathrm{SA}[k]..n])+1, \end{array}$$

concluding the proof.

Example 2. *In the example in* Table 1*, if we look at P*_*DA*_[4] = [1, 2, 4] *and at P*_*DA*_[*LF*(4)] = *P*_*DA*_[14] = [1, 3, 5]*, we have that* Lemma 2 *is verified*.

We now show which elements of the profile of the document array can be extended when performing an LF mapping from a position in a maximal equal-letter run. Those are all the profiles corresponding to occurrences that are all preceded by the same character; that is, the corresponding interval in the suffix array is contained in the maximal equal-letter run. We first recall that given a maximal equal-letter run $\mathrm{BW}T_{T}[s..e]$, the length ℓ of the smallest substring *S* of T such that all occurrences of *S* in T are in $SA_{T}[s..e]$ is given by $\ell =\max(\mathrm{LC}P_{T}[s],\mathrm{LC}P_{T}[e+1])+1$, assuming $\mathrm{LC}P_{T}[n+1]=0$. Note that $SA_{T}[s..e]$ can also contain occurrences of substrings different from *S*.

Lemma 3. *Given a position i in the* BWT *of* T *such that* DA[*i*] = DA[LF(*i*)]*, let* BWT[*s*..*e*] *be the maximal equal-letter run such that s* ≤ *i* ≤ *e,* and *let* ℓ *be the length of the smallest substring S of* T *such that all occurrences of S in* T *are in* $SA_{T}[s..e]$*. Then for all j* = 1, …, *d such that P*_DA_[*i*][*j*] ≥ ℓ, *it holds that P*_DA_[LF(*i*)][*j*] = *P*_DA_[*i*][*j*] + 1.

Proof. The first observation is that if *P*_DA_[*i*][*j*] ≥ ℓ, then there exists a *s* ≤ *k* ≤ *e* such that DA[*k*] = *j*; otherwise, by definition of ℓ and *P*_DA_[*i*][*j*], *P*_DA_[*i*][*j*] < ℓ. Hence, T[SA[k]..n] is preceded by the same character as T[SA[i]..n] because *i* and *k* are in the same BWT run. Therefore, if DA[*k*] = DA[LF[*k*]], we have that lcp(T[SA[LF(k)]..n],T[SA[LF(i)]..n])= lcp(T[SA[k]..n],T[SA[i]..n])+1, which concludes the proof.

Example 3. *In the example in* Table 1*, if we consider i* = 4*, we have that the maximal equal-letter run containing i is* BWT[4..5]; *hence, the smallest substring S of* T *such that all occurrences of S in* T *are in* $SA_{T}[4..5]$ *is* AA, *and its length is given by* ℓ = max (LCP[4], LCP[6]) + 1 = max (0, 1) + 1 = 2*. Looking at P*_*DA*_[4] = [1, 2, 4] *and P*_*DA*_[*LF*(4)] = *P*_*DA*_[14] = [1, 3, 5]*, the only elements of P*_*DA*_[4] *that are not smaller than* two *are P*_*DA*_[4][2] *and P*_*DA*_[4][3], *and we have that P*_*DA*_[14][2] = *P*_*DA*_[4][2] + 1 *and P*_*DA*_[14][3] = *P*_*DA*_[4][3] + 1*, whereas P*_*DA*_[14][1] < *P*_*DA*_[4][1] + 1.

Note that the only case in which we have that DA[*i*] ≠ DA[LF(*i*)] is if the BWT runs is a run of $s. Hence, the above lemma can be applied generally when performing pattern matching queries. We can summarize our solution to Problem 1 in the following theorem.

Theorem 1. *Given a collection* D *of d documents* $D=\{T_{1},T_{2},\ldots ,T_{d}\}$ *over an alphabet of size* σ*, we show how to extend the r*-index *with* O(rd) *additional words to support document listing queries for a pattern S*[1..*m*] *that occurs in ndoc documents in* D *in* $O(m\log\;\log_{w}(\sigma +n/\;r)+ndoc)$*time and* O(rd) *space, where w is the machine word size*. (Query time can be improved to $O(m\log\;\log_{w}\sigma +ndoc)$ by using the approach from Nishimoto et al. 2022.)

Proof. Given a collection D, we store the BWT of the concatenation T of the documents of D, and for all maximal equal-letter runs BWT[*s*..*e*], we store in the positions of *s* and *e* the SA samples SA[*s*] and SA[*e*], as well as the document array profile samples *P*_DA_[LF[*s*]] and *P*_DA_[LF[*e*]].

Let *S*[1..*m*] be a pattern for which we want to compute the list of documents such that *S* occurs in D. After we have processed *S*[*q*..*m*], we have an interval BWT[*s*_*q*_..*e*_*q*_] containing all the occurrences of *S*[*q*..*m*] in T, as well as a profile *P*′ such that for all documents *j*, *P*′[*j*] ≥ (*m* − *q* + 1) if *S*[*q*..*m*] occurs in *T*_*j*_, and *P*′[*j*] < (*m* − *q* + 1) otherwise. Note that the profile is not required to be a document array profile entry for a given position.

If *q* > 1, we now want to extend the match of *S*[*q*..*m*] to *S*[*q* − 1..*m*] and show how we can maintain the invariant of the profile *P*′. We consider two cases. The first case is if BWT[*s*_*q*_..*e*_*q*_] contains either the beginning or the end of a run of the character *S*[*q* − 1]. Here, we can update the interval BWT[*s*_*q*−1_..*e*_*q*−1_] with the standard backward-search and can select as *P*′ the sample of the profile of the document array stored in the run boundary in BWT[*s*_*q*_..*e*_*q*_]. The invariant of *P*′ is preserved by Lemma 1. The second case is when BWT[*s*_*q*_..*e*_*q*_] is completely contained in a run, namely, BWT[*s*_*q*_ − 1] = BWT[*s*_*q*_] = … = BWT[*e*_*q*_] = BWT[*e*_*q*_ + 1]: Then we have that all occurrences of *S*[*q*..*m*] are preceded by the same character; hence by Lemma 3 for all *j* such that *S*[*q*..*m*] occurs in *T*_*j*_, the profile of the document array $P^{\text{″}}$ after the backward step is $P^{\text{″}}[j]=P^{\prime }[j]+1\geq (m-q)$. Furthermore, for all *j* such that *S*[*q*..*m*] does not occur in *T*_*j*_ we have that *P*′[*j*] < (*m* − *q* + 1); hence by Lemma 2, we have that $P^{\text{″}}[j]\leq P^{\prime }[j]+1<(m-q)$. Hence, if for all *j* we set $P^{\text{″}}[j]=P^{\prime }[j]+1$, we have that the invariant requiring that for all documents *j*, $P^{\text{″}}[j]\geq (m-q)$ if *S*[*q* − 1..*m*] occurs in *T*_*j*_ and $P^{\text{″}}[j]<(m-q)$ otherwise is satisfied, concluding the proof.

### Computing the document array profiles

The computation of the document array profiles is performed by scanning the values of BWT, SA, LCP, and DA in a streaming fashion. For all positions *i* = 1, …, *n*, we base the computation of *P*_*DA*_[LF(*i*)] on the observation that given a collection of documents D and a suffix *u* of document *T*_*i*_, the suffix *u* of document *T*_*j*_ with the largest longest common prefix with *u* is the suffix of *T*_*j*_ that either immediately precedes or immediately follows the suffix *u* in SA order. Formally,

Proposition 2. *Given a collection of documents* D *let* T *be the concatenation of its documents. For all indexes i* = 1, …, *n*, *let u*_*i*_ *be the suffix* T[SA[i]..n] *of document* DA[*i*]*. For all documents k* = 1, …, *d*, *let v*_*k*_ *be a suffix* T[SA[j]..n] *of document* DA[*j*] = *k with the largest longest common prefix with u*_*i*_*. We assume w.l.o.g. that v*_*k*_ *is the only suffix with the largest longest common prefix with u*_*i*_*. Then position j corresponding to v*_*k*_ *is either the position of the suffix preceding u*_*i*_ *that is a suffix of document k, namely,* max{*j* < =*i*|DA[*j*] = *k*}*, or the position of the suffix following u*_*i*_ *that is a suffix of document k, namely,* min{*j* > *i*|DA[*i*] = *k*}.

Therefore, we can divide the computation of *P*_*DA*_[LF(*i*)] into two components: the computation of the longest common prefix of T[SA[i]..n] with the suffix of each document immediately preceding the suffix in position *i*, and the longest common prefix of T[SA[i]..n] with the suffix of each document immediately following the suffix in position *i*.

To compute the former, while scanning the values of BWT, SA, LCP, and DA from 1 to *n*, we maintain an auxiliary table Pred[1..*d*][1..σ] such that at step *i*, for all documents *j* = 1, …, *d*, and for all characters *c* = 1, …, σ, Pred[*j*][*c*] stores the length of the longest common prefix value between suffix T[SA[i]..n] and the immediately preceding suffix of document *j* that is preceded by character *c*. The values of Pred[1..*d*][1..σ] can be iteratively computed from the values of Pred[1..*d*][1..σ] at step *i* − 1 as Pred[*j*][*c*] = min (Pred[*j*][*c*], LCP[*i*]), and we set Pred[DA[LF(i)]][BWT[i]]=|T[SA[i]..n]|.

To compute the latter, the intuition is to simulate the maintenance of an auxiliary table equivalent to Pred but for following suffixes and apply an equivalent reasoning for Pred, that is, Succ[1..*d*][1..σ] such that at step *i*, for all documents *j* = 1, …, *d*, and for all characters *c* = 1, …, σ, Succ[*j*][*c*] stores the length of the longest common prefix value between suffix T[SA[i]..n] and the immediately following suffix of document *j* that is preceded by character *c*, if it exists. However, because we are scanning the values of BWT, SA, LCP, and DA from 1 to *n*, the maintenance of such Succ table becomes more difficult. The intuition is that we will build the Succ table for position *i* while evaluating the positions following *i*, and the table will be complete when we have encountered at least one suffix of all documents *j* = 1, …, *d* that is preceded by the character BWT[*i*]. To achieve this, at each step we maintain (1) a queue *LQ* storing tuples of (pos, ch, doc, lcp); (2) a list of incomplete document array profiles that is the same size as *LQ*; and (3) a table *LQC*[1..*d*][1..σ], where for all documents *j* = 1, …, *d*, and for all characters *c* = 1, …, σ, *LQC*[*j*][*c*] stores the number of tuples *t* in *LQ* such that *t*.doc = *j* and *t*.ch = *c*.

At the step *i* we start by inserting the tuple (*i*, BWT[*i*], DA[LF(*i*)], LCP[*i*]) in the queue *LQ*; then we insert Pred[DA[LF[*i*]]][BWT[*i*]] in the list of incomplete document profiles; and we increment the counter in LQC[DA[LF[*i*]]][BWT[*i*]] by one. Then, we update the incomplete document profiles by iterating through all tuples *t* in *LQ* starting from the last inserted element of the queue. While processing tuple *t*, letting ℓ be the length of the longest common prefix between the suffix T[SA[i]..n] and the suffix T[SA[t.pos]..n], we update *P*_*DA*_[LF(*t*.pos)][DA[LF(*i*)]] with max (*P*_*DA*_[LF(*t*.pos)][DA[LF(*i*)]], ℓ) if *t*.ch = BWT[*i*] and *t*.doc ≠ DA[LF(*i*)]. Note that ℓ can be computed while scanning the tuples by initially setting ℓ=|T[SA[i]..n]| and updating ℓ as ℓ = min (ℓ, *t*.lcp).

Finally, we scan the queue *LQ* from the first inserted element to check for finalized, completed document profiles that can be reported. Therefore, while scanning tuple *t*, *P*_*DA*_[LF(*t*.pos)] is complete if all the values in *LQC*[1..*d*][*t*.ch] > 0, meaning we have encountered at least one suffix of all documents *j* = 1, …, *d* that is preceded by the character BWT[*i*]. We then output *P*_*DA*_[LF(*t*.pos)] if it corresponds to a sampled position, namely, if *t*.pos is the beginning or the end of a run. We then decrement the counter in LQC[*t*.doc][*t*.ch] by one and proceed with the next tuple in the queue, and we stop at the first tuple corresponding to an incomplete document profile.

We illustrate an example of the document array profiles in Table 1 along with an example query in Table 2.

### Software availability

The source and experimental codes are available as Supplemental Code and at GitHub (https://github.com/oma219/docprofiles and https://github.com/oma219/docprof-experiments, respectively). The source code is also available at Zenodo (https://doi.org/10.5281/zenodo.7942523).

### Competing interest statement

The authors declare no competing interests.

## Supplementary Material

Supplement 1
